# Supplementary material for: Change in confidence in public health entities among US adults between 2020–2024
Source: PLOS Glob Public Health. 2025 Jun 26;5(6):e0004747. doi: 10.1371/journal.pgph.0004747 (PMC12200701; doi:10.1371/journal.pgph.0004747)
Supplement: S1 Table — (DOCX) [file pgph.0004747.s001.docx]

**Supplementary Material for** “Change in confidence in public health entities among US adults, 2020-2024

**S1 Table.** Change in weighted percentage reporting high confidence (2020 – 2024*)

| **Entity** | **Survey 1** | **Survey 2** | **Proportion Change (95% CI)** | **p-value** |
| --- | --- | --- | --- | --- |
| Your own doctor | 0.79 | 0.77 | 0.02 (-0.03 - 0.07) | 0.378 |
| Local health department | 0.67 | 0.62 | 0.05 (-0.001 - 0.10) | 0.056 |
| State health department | 0.68 | 0.67 | 0.01 (-0.04 - 0.06) | 0.697 |
| Centers for Disease Control and Prevention | 0.82 | 0.68 | 0.14 (0.09 - 0.19) | <0.001 |
| Food and Drug Administration | 0.58 | 0.52 | 0.06 (0.01 - 0.11) | 0.028 |
| The White House | 0.29 | 0.36 | -0.07 (-0.12 - -0.02) | 0.006 |
| Department of Health and Human Services | 0.61 | 0.47 | 0.14 (0.09 - 0.19) | <0.001 |
| National Institute of Health | 0.74 | 0.54 | 0.2 (0.15 - 0.25) | <0.001 |
| Professional medical organizations (i.e. AMA) | 0.75 | 0.63 | 0.12 (0.07 - 0.17) | <0.001 |
| **Entity** | **Survey 1** | **Survey 3** | **Proportion Change (95% CI)** | **p-value** |
| Your own doctor | 0.79 | 0.68 | 0.11 (0.07 - 0.16) | <0.001 |
| Local health department | 0.67 | 0.54 | 0.13 (0.08 - 0.18) | <0.001 |
| State health department | 0.68 | 0.52 | 0.16 (0.11 - 0.21) | <0.001 |
| Centers for Disease Control and Prevention | 0.82 | 0.56 | 0.26 (0.22 - 0.3) | <0.001 |
| Food and Drug Administration | 0.58 | 0.46 | 0.12 (0.07 - 0.17) | <0.001 |
| The White House | 0.29 | 0.32 | -0.03 (-0.08 - 0.17) | 0.212 |
| Department of Health and Human Services | 0.61 | 0.48 | 0.13 (0.08 - 0.18) | <0.001 |
| National Institute of Health | 0.74 | 0.49 | 0.25 (0.2 - 0.3) | <0.001 |
| Professional medical organizations (i.e. AMA) | 0.75 | 0.49 | 0.26 (0.21 - 0.31) | <0.001 |
| **Entity** | **Survey 1** | **Survey 4** | **Proportion Change (95% CI)** | **p-value** |
| Your own doctor | 0.79 | 0.73 | 0.06 (0.02 - 0.1) | 0.007 |
| Local health department | 0.67 | 0.73 | -0.06 (-0.1 - -0.01) | 0.012 |
| State health department | 0.68 | 0.57 | 0.11 (0.06 - 0.16) | <0.001 |
| Centers for Disease Control and Prevention | 0.82 | 0.6 | 0.22 (0.18 - 0.26) | <0.001 |
| Food and Drug Administration | 0.58 | 0.55 | 0.03 (-0.02 - 0.08) | 0.247 |
| The White House | 0.29 | 0.39 | -0.1 (-0.15 - -0.05) | <0.001 |
| Department of Health and Human Services | 0.61 | 0.54 | 0.07 (0.02 - 0.12) | 0.007 |
| National Institute of Health | 0.74 | 0.57 | 0.17 (0.12 - 0.22) | <0.001 |
| Professional medical organizations (i.e. AMA) | 0.75 | 0.54 | 0.21 (0.15 - 0.26) | <0.001 |
| **Entity** | **Survey 2** | **Survey 3** | **Proportion Change (95% CI)** | **p-value** |
| Your own doctor | 0.77 | 0.68 | 0.09 (0.04 - 0.14) | <0.001 |
| Local health department | 0.62 | 0.54 | 0.08 (0.03 - 0.13) | 0.002 |
| State health department | 0.67 | 0.52 | 0.15 (0.10 - 0.20) | <0.001 |
| Centers for Disease Control and Prevention | 0.68 | 0.56 | 0.12 (0.07 - 0.17) | <0.001 |
| Food and Drug Administration | 0.52 | 0.46 | 0.06 (0.01 - 0.11) | 0.025 |
| The White House | 0.36 | 0.32 | 0.04 (-0.01 - 0.09) | 0.113 |
| Department of Health and Human Services | 0.47 | 0.48 | -0.01 (-0.06 - 0.04) | 0.709 |
| National Institute of Health | 0.54 | 0.49 | 0.05 (-0.003 - 0.10) | 0.064 |
| Professional medical organizations (i.e. AMA) | 0.63 | 0.49 | 0.14 (0.09 - 0.19) | <0.001 |
| **Entity** | **Survey 2** | **Survey 4** | **Proportion Change (95% CI)** | **p-value** |
| Your own doctor | 0.77 | 0.73 | 0.04 (-0.01 - 0.09) | 0.085 |
| Local health department | 0.62 | 0.73 | -0.11 (-0.16 - -0.06) | <0.001 |
| State health department | 0.67 | 0.57 | 0.1 (0.05 - 0.15) | <0.001 |
| Centers for Disease Control and Prevention | 0.68 | 0.6 | 0.08 (0.03 - 0.13) | 0.02 |
| Food and Drug Administration | 0.52 | 0.55 | -0.03 (-0.08 - 0.02) | 0.261 |
| The White House | 0.36 | 0.39 | -0.03 (-0.08 - 0.21) | 0.247 |
| Department of Health and Human Services | 0.47 | 0.54 | -0.07 (-0.12 - -0.02) | 0.009 |
| National Institute of Health | 0.54 | 0.57 | -0.03 (-0.08 - 0.02) | 0.262 |
| Professional medical organizations (i.e. AMA) | 0.63 | 0.54 | 0.09 (0.04 - 0.13) | 0.001 |
| **Entity** | **Survey 3** | **Survey 4** | **Proportion Change (95% CI)** | **p-value** |
| Your own doctor | 0.68 | 0.73 | -0.05 (-0.1 - -0.01) | 0.031 |
| Local health department | 0.54 | 0.73 | -0.19 (-0.24 - -0.14) | <0.001 |
| State health department | 0.52 | 0.57 | -0.05 (-0.1 - -0.001) | 0.047 |
| Centers for Disease Control and Prevention | 0.56 | 0.6 | -0.04 (-0.09 - 0.01) | 0.109 |
| Food and Drug Administration | 0.46 | 0.55 | -0.09 (-0.14 - -0.04) | <0.001 |
| The White House | 0.32 | 0.39 | -0.07 (-0.12 - -0.02) | 0.004 |
| Department of Health and Human Services | 0.48 | 0.54 | -0.06 (-0.11 - -0.01) | 0.018 |
| National Institute of Health | 0.49 | 0.57 | -0.08 (-0.13 - -0.3) | 0.002 |
| Professional medical organizations (i.e. AMA) | 0.49 | 0.54 | -0.05 (-0.1 - -0.0003) | 0.049 |
| *Survey *Survey 1 – February 2020; Survey 2 – May 2020; Survey 3 – June 2022; Survey 4 – October 2024 | | | | |
