# Supplementary material for: Change in confidence in public health entities among US adults between 2020–2024
Source: PLOS Glob Public Health. 2025 Jun 26;5(6):e0004747. doi: 10.1371/journal.pgph.0004747 (PMC12200701; doi:10.1371/journal.pgph.0004747)
Supplement: Attachment — Submitted filename: Confidence Brief_Response to Reviewers_2025 05 08.docx [file pgph.0004747.s003.docx]

**Point-by-point response for** “Change in confidence in public health entities among US adults between 2020-2024”

**Author response:** We would like to thank the reviewers for their thoughtful and constructive review of our manuscript. We have addressed all comments in the table below, as well as journal requirements. Thank you!

**Journal Requirements:**

1. In the online submission form, you indicated that De-identified data can be provided upon reasonable request.

All PLOS journals now require all data underlying the findings described in their manuscript to be freely available to other researchers, either

1. In a public repository,

2. Within the manuscript itself, or

3. Uploaded as supplementary information.

This policy applies to all data except where public deposition would breach compliance with the protocol approved by your research ethics board. If your data cannot be made publicly available for ethical or legal reasons (e.g., public availability would compromise patient privacy), please explain your reasons by return email and your exemption request will be escalated to the editor for approval. Your exemption request will be handled independently and will not hold up the peer review process but will need to be resolved should your manuscript be accepted for publication. One of the Editorial team will then be in touch if there are any issues.

Response: We have uploaded the relevant data to GitHub, available publicly here: https://github.com/OmerResearchGroup/Change-in-confidence-in-public-health-entities-among-US-adults-between-2020-2024

2. We notice that your supplementary tables are included in the manuscript file. Please remove them and upload them with the file type 'Supporting Information'. Please ensure that each Supporting Information file has a legend listed in the manuscript after the references list.

Response: **A separate supplementary file has been uploaded.**

3. Please review your reference list to ensure that it is complete and correct. If you have cited papers that have been retracted, please include the rationale for doing so in the manuscript text or remove these references and replace them with relevant current references. Any changes to the reference list should be mentioned in the rebuttal letter that accompanies your revised manuscript. If you need to cite a retracted article, indicate the article’s retracted status in the References list and include a citation and full reference for the retraction notice.

Response: **We have also added two clarifying references, a recently published article on the overall results of our 2024 mpox survey (Melchinger et al., #4) and an article describing how CloudResearch recruits demographically representative samples for research studies (Moss and Litman, #5).**

**Reviewer 1**

Comment: Your methods section is well organized and provides a clear overview of survey development, analysis, and ethical considerations. However, there are a few areas where clarity, consistency, and detail can be improved.

Strengths: the sections flow smoothly. You specify the use of CloudResearch and Qualtrics, as well as the survey timeframe and incentives Appropriate statistical analysis methods – use of survey weighting, confidence intervals, and p-values is correctly described. IRB approvals and ethical considerations are well-documented.

| **No.** | **Comment** | **Response** |
| --- | --- | --- |
| 1 | **Clarity of survey design (cross sectional, longitudinal, or sampling?)** The sentence "Between February 2020 and October 2024, we conducted four surveys assessing confidence in public health entities..." implies a continuous process, but surveys were conducted at four distinct time points (not continuously). Specify whether the surveys were discrete cross-sectional assessments or an ongoing study. | Thank you for this comment. As you say, the surveys were conducted at four distinct timepoints: they were discrete cross-sectional assessments rather than one long study.  We have clarified the sentence as follows in the Methods:  “Between February 2020 and October 2024, we conducted four discrete, cross-sectional surveys assessing confidence in public health entities among US adults [1-4].” |
| 2 | **Standardize date formatting for consistency.**  "Participants were recruited from February 4-7, 2020, for Survey 1, from May 6-7, 2020, for Survey 2, from June 8-13, 2022, for Survey 3, and from September 20-27, 2024, for Survey 4." The mix of different prepositions and punctuation makes this harder to read. Consider "Survey 1 was conducted from February 4–7, 2020; Survey 2 from May 6–7, 2020; Survey 3 from June 8–13, 2022; and Survey 4 from September 20–27, 2024." | The sentence has been updated as suggested:  “Survey 1 was conducted from February 4-7, 2020; Survey 2 from May 6-7, 2020; Survey 3 from June 8-13, 2022; and Survey 4 from September 20-27, 2024.” |
| 3 | **Were participants randomly selected?** Stratified? Quota-based? | Participants were recruited from CloudResearch’s survey system in accordance with quotas indicated by the American Community Survey (ACS) census estimates. Within each quota, participants were randomly selected.  We have clarified the sentence as follows:  “A representative sample was recruited from CloudResearch’s pre-existing survey panels, which consist of individuals recruited by word-of-mouth and advertising to take surveys. Respondents were recruited according to demographic quotas proportional to the U.S. Census; within each quota, respondents were selected randomly.[4]” |
| 4 | "Survey weights were calculated based on age, gender, and race estimations sourced from the American Community Survey (ACS) 2022." What about education level, geographic region, or income? Why were they excluded? | We did not weight our survey results by income because we did not collect information on participant income in our survey; similarly, information on geographic region was not asked in all surveys. Information on education level stratified by age, sex, and race, was not readily available in the ACS estimates. |
| 5 | **Strengthen survey weighting and statistical analysis description.** "Significance was determined as anything with a p-value less than an alpha of 0.05." sounds too informal—use "statistical significance was defined as p <0.05." | We have clarified the sentence as suggested:  “Statistical significance was defined as p<0.05.” |
| 6 | **Improve ethical statement to ensure clarity on data privacy & consent.** "Participants were advised at the start of the survey that by continuing with the questionnaire, they were consenting to participating in the study." Consider "consenting to participate." | We have made this change:  “Respondents were advised at the start of the survey that by continuing with the questionnaire, they were consenting to study participation.” |
| 7 | **Address potential metadata collection concerns for better confidentiality assurances.** The statement "No personal data was collected from respondents." is good, but does it include IP addresses, cookies, or metadata? Qualtrics automatically collect metadata (e.g., location, device type). If this data was not stored or used, clarify this to strengthen data privacy assurances. | Surveys were delivered via anonymous link, and no metadata including IP address, location, or device type was stored or used in Qualtrics.  The following sentence was added for clarity:  “No personal data was collected from respondents; survey metadata, included IP address, device type, and location, were not stored or used.” |

**Reviewer 2**

Comment: The topic is interesting. The manuscript has a good flow and is written in brief and an interesting way. I have a few suggestions to consider.

| **No.** | **Comment** | **Response** |
| --- | --- | --- |
| 1 | **Was the study population the same for all four surveys?** While the method section describes the participants were recruited from pre-existing panels in the CloudResearch survey system, but do not explicitly state whether the same individuals participated in all four surveys or if each survey comprised a new, independent sample drawn from the same pool. | The study population was not the same for any of the surveys, but all study populations were recruited in the same way to ensure representativeness. Participants were recruited through CloudResearch’s survey system in accordance with quotas indicated by the American Community Survey (ACS) census estimates, and randomly within each quota (see earlier comment/response).  We have clarified this point as follows:  “Distinct representative study populations were recruited from the same panels for all studies. Survey 1 was conducted from February 4-7, 2020; Survey 2 from May 6-7, 2020; Survey 3 from June 8-13, 2022; and Survey 4 from September 20-27, 2024.” |
| 2 | **What was the sample size of all four surveys?** | Samples sizes for each survey are as follows:  Survey 1: 718  Survey 2: 672  Survey 3: 856  Survey 4: 828  These numbers are available in papers published about the individual studies, cited as references 1-4. We have also added them in the methods as follows:  “Sample sizes for Surveys 1-4 were 718, 672, 856, and 828, respectively.” |
| 3 | **The IRB permission from two different universities also create slight confusion.** For the first three survey (2020 and 2022) Institutional Review Board permission taken from Yale University while for 4th survey (2024) taken from University of Texas. These two universities are located in different states, and their residents may differ in perception regards public entities. Consider highlighting it in limitation section, if the survey population is different in these four surveys. | Thanks for noting this – the IRBs were granted by two different institutions because our research group moved to UTSW in 2023 (whereas we were at Yale during the first three studies). However, the study populations were not affected by which state we were based in – by working with CloudResearch to recruit a census-matched study, we sought to administer the survey to a representative sample of the entire US population. |
| 4 | **In methods section last para –** ‘Among disease-specific questions’, can you explain what you mean by this, I could not see its relevance in the current study. Kindly rewrite the sentence for clarity (confidence in public health entities repeated twice in same sentence) | Thank you for noting this: we have clarified the sentence as follows:  “Surveys 1 and 2 asked questions about to the COVID-19 pandemic, while Surveys 3 and 4 asked questions around the 2022 and 2024 mpox outbreaks. In addition to these disease-specific questions, we asked participants a series of questions about their perceptions of public health entities, including who they thought should lead the US response to infectious disease outbreaks, and how they would rate their confidence in different public health entities.” |
